# Observations with Antitetanus Serum in the Monkey

**Published:** 1918-01

**Authors:** 


***Observations with Antitetanus Serum in the Monkey.*** By C. S. SHERRINGTON, Professor of Physiology, Oxford University. Abs. from the *Lancet*, Dec. 29, 1917.

The experiments reported by S. were carried out in connection with the work of the Tetanus Committee. Monkeys of various species were used for the experiments, but there appeared to be no difference among them as to susceptibilily.

Each animal was inoculated in the same way with a like dose of toxin. Upon the appearance of tetanus symptoms like doses of antitoxin were injected in the monkeys, but by the following various routes : (1) subcutaneous, (2) intramuscular, (3) intravenous, (4) lumbar intrathecal, (5) bulbar intrathecal (6), cerebral subdural. The toxin and part of the antitoxin were supplied by the Lister Institute; the remainder of the antitoxin by Burroughs and Wellcome. The dose of toxin was 26 mgm. per kilo. monkey : the dose of antitoxin, 2000 U. S. A. units per kilo. monkey.

By the subcutaneous route S. obtained recoveries of 2 out of 25 monkeys : by the intramuscular route, 3 out of 25 : by the intravenous route, 7 out of 25 : by the lumbar intrathecal, 14 out of 25 : and by the bulbar intrathecal, 13 out of 20. None were saved by the cerebral subdural route.

A few experiments were tried (on 10 monkeys) to determine the duration of prophylaxis. Control monkeys without prophylaxis died in from 4 to 5 days. Those treated prophylactically were prevented from having general tetanus from toxin administered as late as 11 days after the preventive treatment. Local symptoms, however, appeared.

